# Supplementary material for: The Nup2 meiotic-autonomous region relieves inhibition of Nup60 to promote progression of meiosis and sporulation in Saccharomyces cerevisiae
Source: Genetics. 2022 Mar 18;221(1):iyac045. doi: 10.1093/genetics/iyac045 (PMC9071577; doi:10.1093/genetics/iyac045)
Supplement: iyac045_Supplemental_Material [file iyac045_supplemental_material.pdf]

Supplemental Figures, Tables, Methods, and References for Komachi and Burgess, 2022

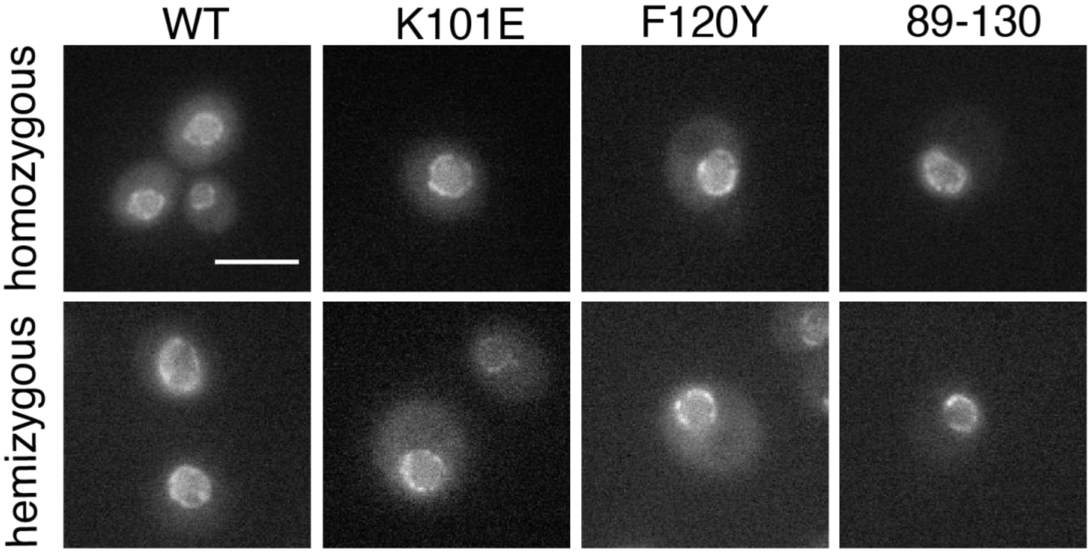

Figure S1. Effect of hemizygosity on localization of mar(K101E), mar(F120Y), and mar(89-130) GFP fusions. Top panels show fluorescent images of the homozygous strains, bottom panels show hemizygous (*mar/nup2Δ*) strains. The MAR allele depicted is indicated at the top of each column. The scale bar in the wild-type MAR-GFP panel represents 5 μm.

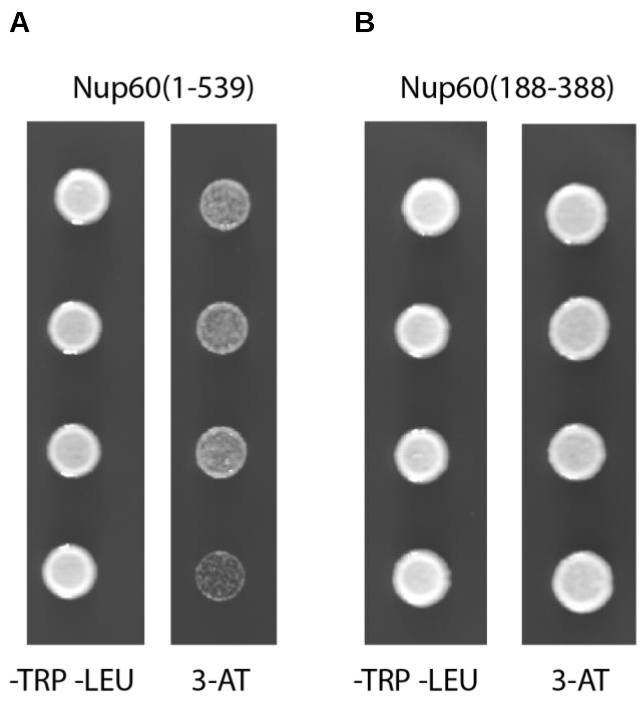

Figure S2. Biological replicates of the MAR interacting with full-length Nup60(1-539) or Nup60(188-388) in the Y2H assay. (A) A pGAL1-HIS3 strain was transformed with *MAR-GAL4DBD* and *NUP60(1-539)-GAL4AD* plasmids. Cultures grown from four independent transformants were spotted onto -Trp -Leu (left) and -Trp -Leu -His + 5 mM 3-aminotriazole plates (right, 3-AT). (B) Same as (A), except with *NUP60(188-388)-GAL4AD* instead of *NUP60(1-539)-GAL4AD*.

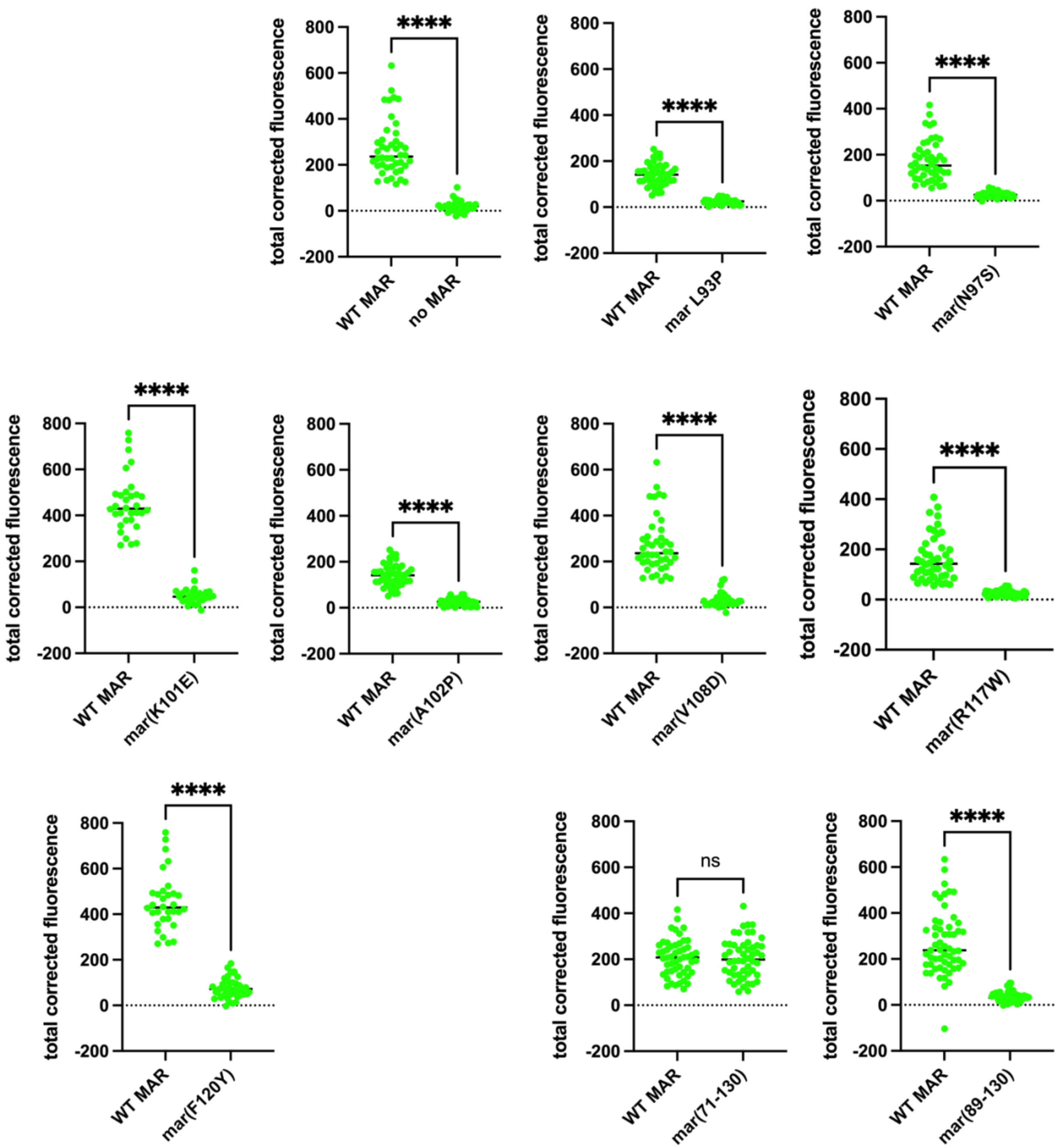

Figure S3. Quantitation of fluorescence from meiotic chromosome spreads from strains expressing wild-type or mutant versions of the MAR fused to GFP. Total corrected fluorescence was measured as described in the Methods. Each panel shows results for two combined sets of mutant and wild-type spreads stained on the same day. P < 0.0001 for all mutant/WT comparisons using a two-tailed t-test, except for mar(71-130), in which P = 0.95.

A

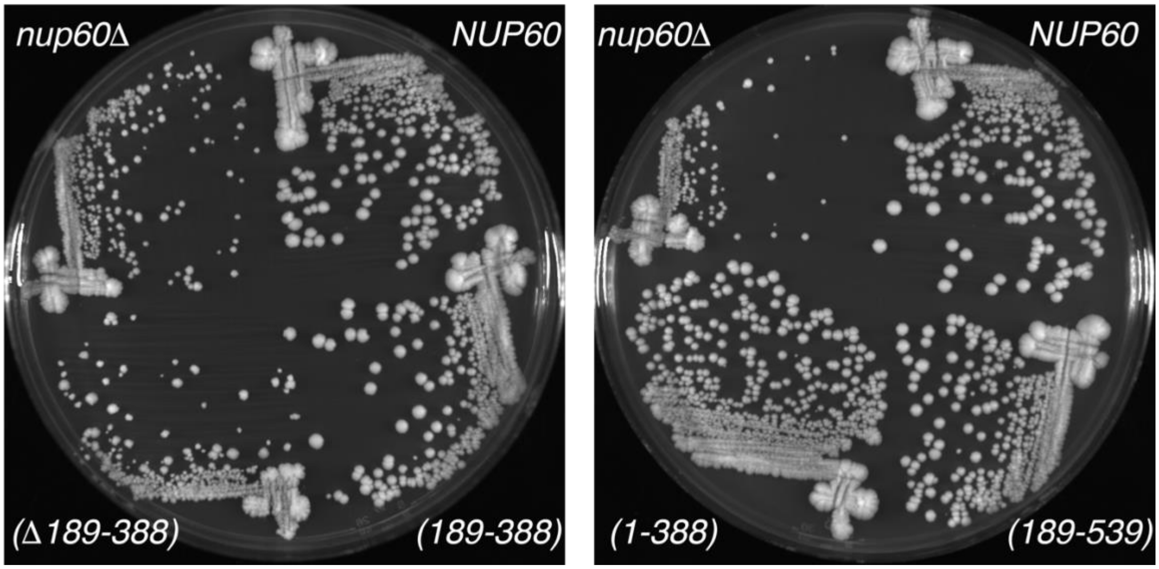

B

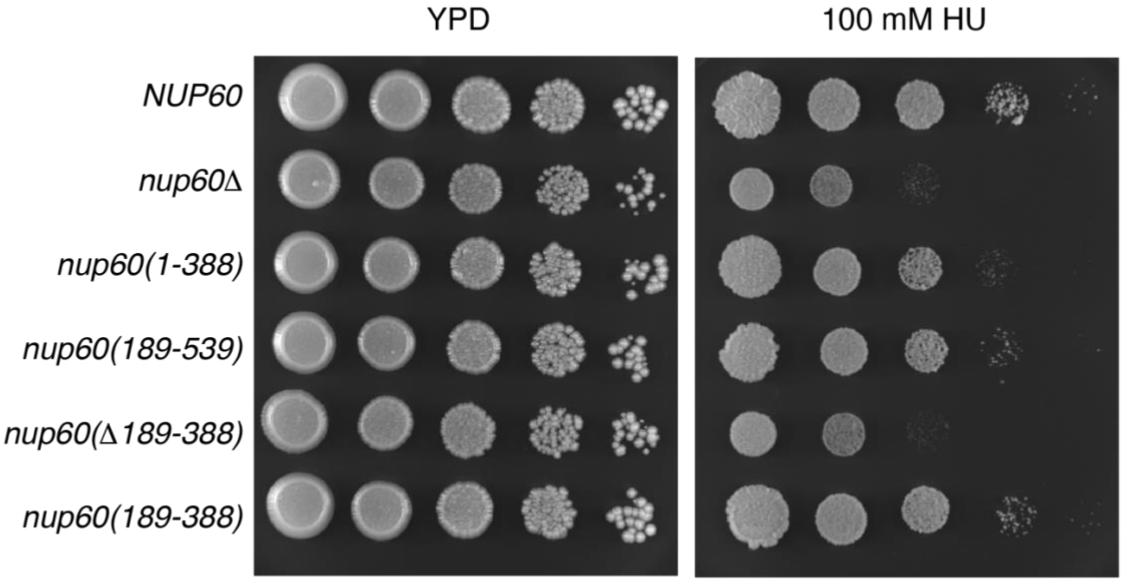

Figure S4. Complementation of the growth defect and HU sensitivity by *NUP60* fragments. (A) Strains carrying the indicated alleles of *NUP60* were streaked onto YPD plates and grown for 48 hours at 30˚C. The *nup60Δ* and *nup60(Δ189-388)* strains exhibit poor growth, forming smaller colonies with a nibbled morphology. (B) Strains were grown to mid-log phase in liquid YPD and diluted to an OD_{600} of 1. Serial 10-fold dilutions were spotted onto YPD (left) or YPD plus 100 mM hydroxyurea (right) and grown at

30˚C for 48 hours and 96 hours, respectively. For both (A) and (B), the strains are isogenic to the wild-type (*NUP60*) strain: *NUP60* (SBY1903), *nup60Δ* (SBY5217), *nup60(1-388)* (SBY6423), *nup60(189-539)* (SBY6426), *nup60(Δ189-388)* (SBY6429), *nup60(189-388)* (SBY6420).

A

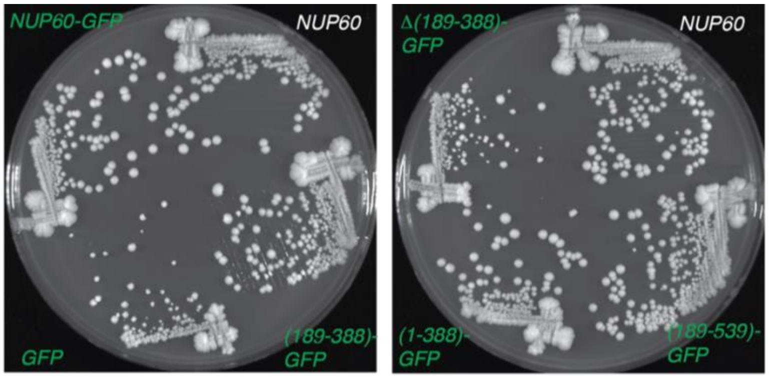

B

Relevant genotype	Growth	Sporulation efficiency
NUP60-GFP	normal	72 ± 7
GFP	poor	15 ± 5
(189-388)-GFP	normal	20 ± 6
(1-388)-GFP	normal	68 ± 7
(189-539)-GFP	normal	15 ± 3
(Δ189-388)-GFP	poor	16 ± 4

Figure S5. Complementation of growth and sporulation by truncated Nup60-GFP fusions. (A) Growth was assessed by streaking strains onto YPD plates and growing at 30˚C for 48 hours. Poorly growing strains formed smaller colonies with a nibbled morphology. All strains are isogenic to the wild-type (*NUP60*) strain: *NUP60* (SBY1899), *NUP60-GFP* (SBY6304), *GFP* (SBY6307), *nup60(189-388)-GFP* (SBY6310), *nup60(1-388)* (SBY6313), *nup60(189-539)* (SBY6316), *nup60(Δ189-388)-GFP* (SBY6382). (B) The growth column is a summary of the results in (A). The strains used to assay sporulation efficiency are in an *ndj1* background and are all isogenic to the *NUP60-GFP* strain: *NUP60-GFP* (SBY6320), *GFP* (SBY6323), *nup60(189-388)-GFP* (SBY6326), *nup60(1-388)* (SBY6329), *nup60(189-539)* (SBY6332), *nup60(Δ189-388)-GFP* (SBY6335).

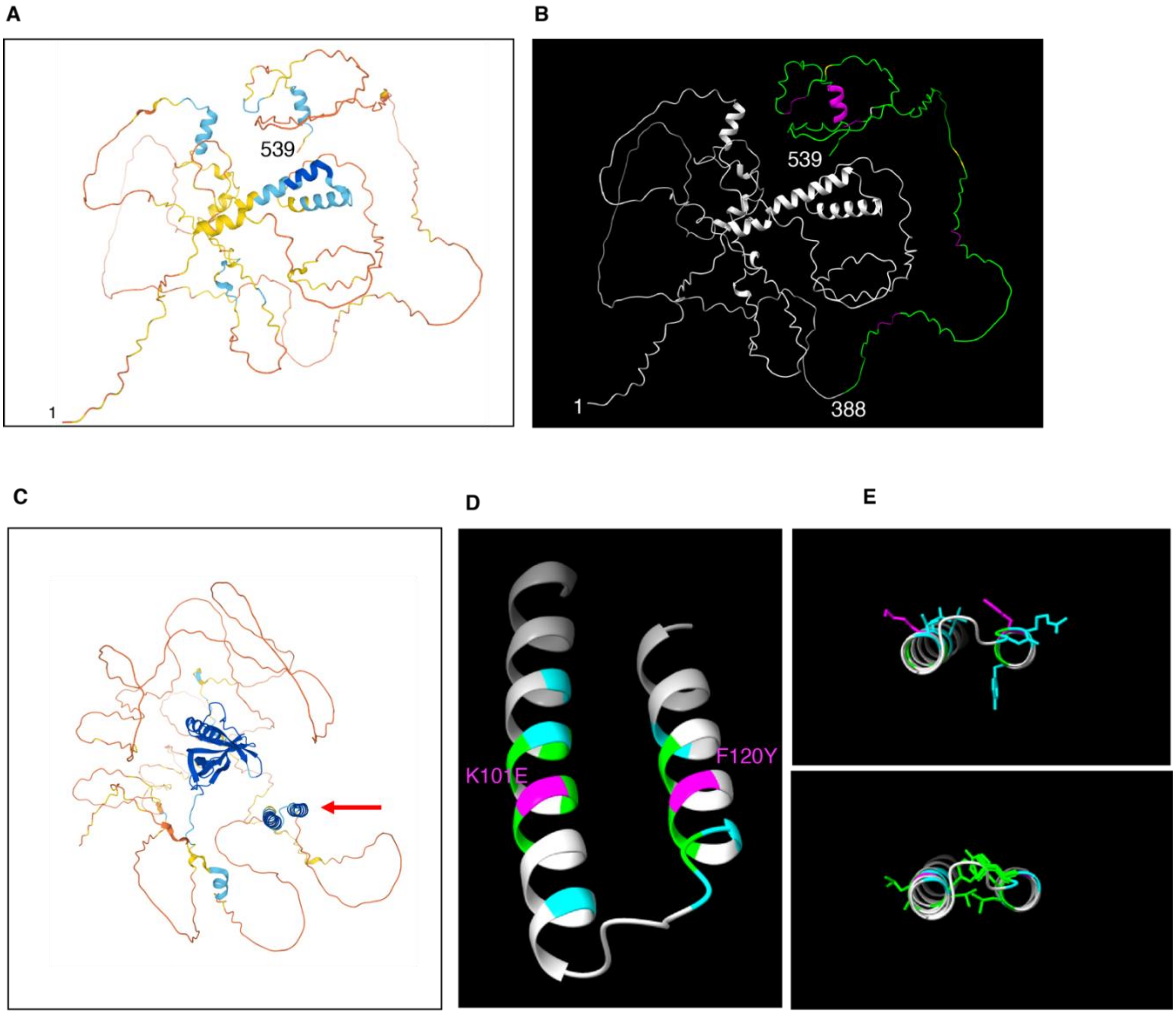

Figure S6. AlphaFold predictions of the structures of Nup60 and Nup2. (A) Nup60 predicted structure from the AlphaFold v2.0 database updated on 1 July 2021 (Jumper *et al.* 2021). Regions are color-coded according to AlphaFold confidence scores based on the local distance difference test (LDDT) (Mariani *et al.* 2013): Very high (pLDDT > 90); Confident (90 > pLDDT > 70); Low (70 > pLDDT > 50); Very low (pLDDT < 50). The first and last amino acids are numbered. (B) Nup60 predicted structure, with the region sufficient for sporulation function (aa 1-388) colored gray and the region containing the inhibitory domain (aa 389-539) colored green. A predicted helix near the extreme C-terminus is colored magenta; the FxF repeats (aa 399-401, 427-429, 469-471, 509-511), purple;

SUMOylation sites (aa 440, 442, 505), yellow; a known acetylation site (aa 467), white. (C) AlphaFold v2.0 prediction of the structure of Nup2, with regions color-coded according to confidence scores described in (A). The MAR (indicated by the red arrow) and Ran GTPase binding domain are the only two regions where structure can be predicted with high confidence. (D) Blow-up of the helices that make up the MAR, rotated approximately 90˚ around the x-axis with respect to (C). The positions of the mutations are marked by different colors: mutations that do not disrupt localization to the nuclear envelope (K101 and F120) are colored magenta; mutations that disrupt localization but not binding to full-length Nup60 in Y2H, cyan; mutations that disrupt localization and all Y2H interaction, green. (E) Helices of the MAR in the same orientation as in (C), with side chains shown. The top panel highlights mutations that do not disrupt binding to full-length Nup60 in the Y2H assay; the bottom panel highlights mutations that disrupt Y2H to full-length Nup60. Images in B, D, and E were created using AlphaFold v2.0 coordinates and UCSF ChimeraX version 1.2.5 (Pettersen *et al.* 2021).

```
                               *  *** ***     *      **  **  *  *
S. cerevisiae Nup2      89  SNSRLKALNLQFKAKVDDLVLGKPLADLRPLFTRYELYIKNI     133
H. sapiens Nup50       144 AYHKQLAALNCSVRDWIVKHVNTNPLCDLTPIFKDYEKYLANIEQQ 189
M. musculus Nup50      144 AYHKQLAGLNCSVRDWIVKHVNTNPLCDLTPIFKDYERYLATIEKQ 189
X. laevis Nup50        144 EYNKQLTSLNCSVRDWIVKHVNANPLCDLTPIFKDYERKLSAIEQK 189
```

Figure S7. Fragment of *Xenopus laevis* Nup50 sufficient for binding to Nup153 and MEL28/ELYS (Holzer *et al.* 2021) aligned with mammalian Nup50s and the MAR from *S. cerevisiae* Nup2.

A

Relevant genotype	Sporulation efficiency
3HA-NUP60	82 ± 8
3HA-NUP60 ndj1	22 ± 4
3HA-NUP60 nup2	75 ± 5
3HA-NUP60 ndj1 nup2	14 ± 2

B

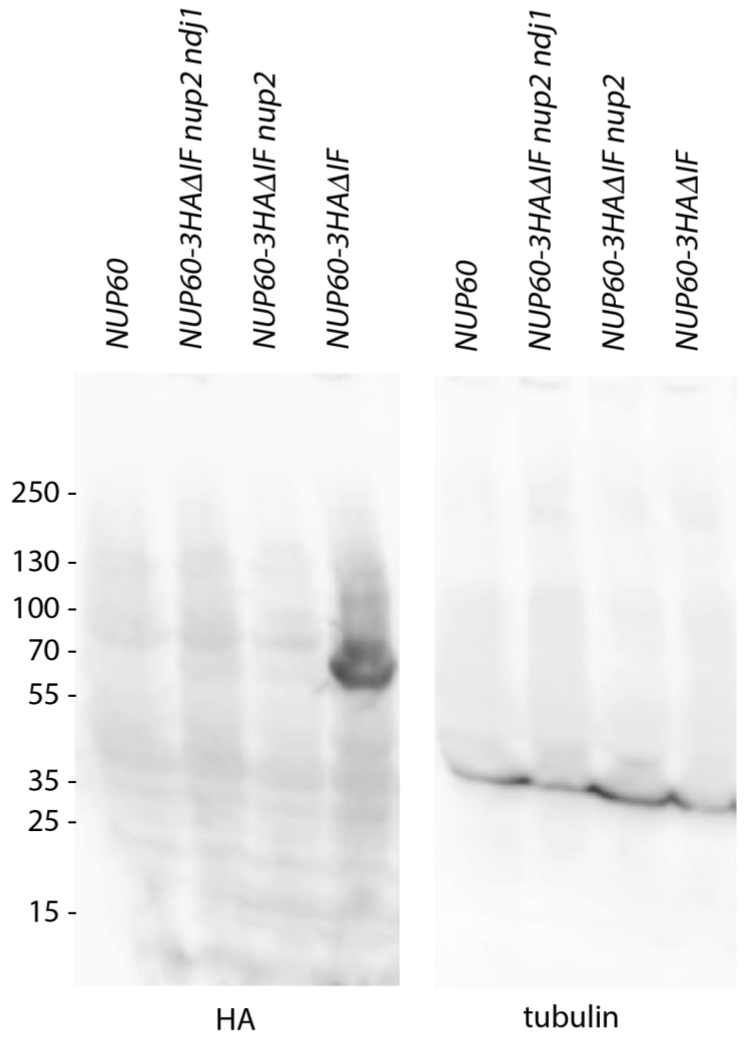

Figure S8. Disruption of normal Nup60 function by HA tagging at the N- or C-terminus. (A) Sporulation efficiency of *3HA-NUP60* strains. N-terminally tagged Nup60 is unable to promote sporulation in the absence of Ndj1. (B) Western blot of *NUP60* or *NUP60-3HA* strains probed with an antibody against HA (left) or β-tubulin (right). The linker between Nup60 and the HA tag has been modified to remove two amino acids known to destabilize HA tagged proteins in yeast (Saiz-Baggetto *et al.* 2017). The fusion is strongly expressed in wild-type cells but cannot be detected in extracts from *nup2* strains.

Unlike *nup60Δ nup2Δ* strains, the *NUP60-3HA nup2* strains grow well, suggesting that the Nup60-HA fusion is being partially degraded, leaving enough Nup60 to support cell growth. It is unclear why Nup60-3HA is only unstable in the absence of Nup2.

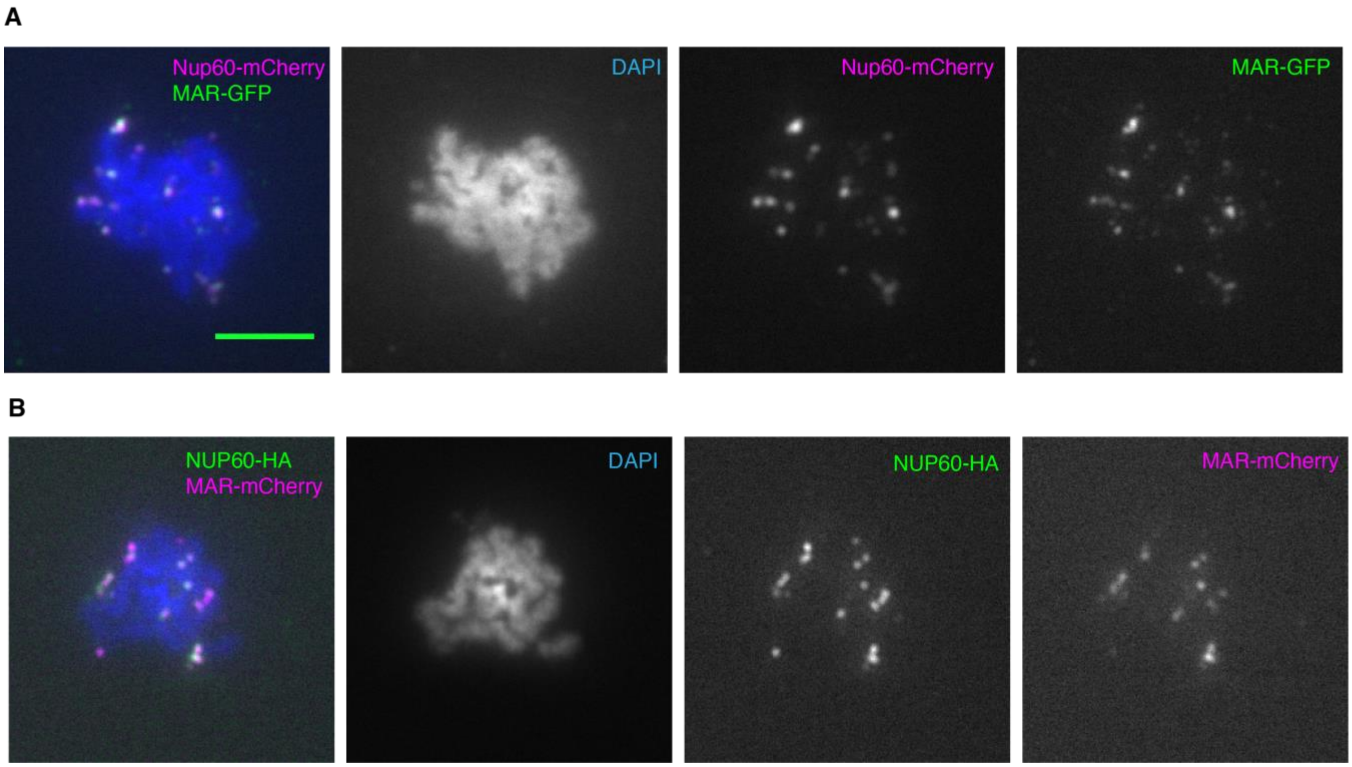

Figure S9. Binding of meiotic chromosomes by Nup60-mCherry and Nup60-3HA. (A) Representative meiotic chromosome spreads from a strain expressing Nup60-mCherry and MAR-GFP (SBY6481). Left to right are the merged image and images from the DAPI, TRITC, and FITC channels. (B) Representative meiotic chromosome spreads from a strain expressing Nup60-3HA and MAR-mCherry (SBY6410). Left to right are the merged image and images from the DAPI, FITC, and TRITC channels. The GFP fusions were detected using a polyclonal antibody to GFP and an Alexa Fluor 488-conjugated secondary antibody. Nup60-mCherry was detected using a polyclonal antibody to mCherry and an Alexa Fluor 594-conjugated secondary. Nup60-3HA was detected using a polyclonal antibody to HA and an Alexa Fluor 594-conjugated secondary. The scale bar in the upper left panel represents 5 μm.

Table S1. Yeast strains used in this study

Strain	Genotype	Background	Figure Panel	References
SBY6259	*MATa ho::hisG leu2::hisG ura3::hisG GAL3(?) his4::LEU2-(NBam) nup2(Δ51-175) ndj1::TRP1*	SK1	Figure 1B (Screen)	
SBY6260	*MATalpha ho::hisG leu2::hisG ura3(ΔSma-PstI) HIS4::LEU2-(NBam) nup2::KanMx ndj1::KanMx*	SK1	Figure 1B (Screen)	
SBY1899	*MATalpha ho::hisG leu2::hisG ura3(ΔSma-Pst) his4-X::LEU2-(NBam)-URA3*	SK1	Wild-type parent of SBY1903	(Chu *et al.* 2017)
SBY1900	*MATa ho::hisG leu2::hisG ura3(ΔSma-Pst) HIS4::LEU2-(NBam)*	SK1	Wild-type parent of SBY1903	(Chu *et al.* 2017)
SBY1903	*MATa ho::hisG leu2::hisG ura3(ΔSma-Pst) HIS4::LEU2-(NBam)*<br>*MATalpha ho::hisG leu2::hisG ura3(ΔSma-Pst) his4-X::LEU2-(NBam)-URA3*	SK1	Wild-type diploid strain	(Chu *et al.* 2017)
SBY6062	*MATa ho::hisG leu2::hisG ura3(ΔSma-Pst) HIS4::LEU2-(NBam) ndj1::KanMx nup2(51-175)-GFP::CaURA3*<br>*MATalpha ho::hisG leu2::hisG ura3(ΔSma-Pst) his4-X::LEU2-(NBam) ndj1::KanMx nup2(51-175)-GFP::CaURA3*	SK1	Table 1 (sporulation, *ndj1* background) wt MAR control	
SBY6062*	*MATa ho::hisG leu2::hisG ura3(ΔSma-Pst) HIS4::LEU2-(NBam) ndj1::KanMx nup2(51-175)*-GFP::CaURA3*<br>*MATalpha ho::hisG leu2::hisG ura3(ΔSma-Pst) his4-X::LEU2-(NBam)-URA3 ndj1::KanMx nup2(51-175)*-GFP::CaURA3*	SK1	Table 1 (sporulation, *ndj1* background) Point mutant versions of SBY6062	
SBY6059	*MATa ho::hisG leu2::hisG ura3(ΔSma-Pst) HIS4::LEU2-(NBam) ndj1::KanMx nup2(Δ1-720)-GFP::CaURA3*<br>*MATalpha ho::hisG leu2::hisG ura3(ΔSma-Pst) his4-X::LEU2-(NBam)-URA3 ndj1::KanMx nup2(Δ1-720)-GFP::CaURA3*	SK1	Table 1 (sporulation, *ndj1* background) GFP (no MAR) control	
SBY6065	*MATa ho::hisG leu2::hisG ura3(ΔSma-Pst) HIS4::LEU2-(NBam) ndj1::KanMx nup2(71-130)-GFP::CaURA3*	SK1	Table 1 (sporulation, *ndj1* background)	

	MATalpha ho::hisG leu2::hisG ura3(ΔSma-Pst) his4-X::LEU2-(NBam)-URA3 ndj1::KanMx nup2(71-130)-GFP::CaURA3			
SBY6266	*MATa ho::hisG leu2::hisG ura3(ΔSma-Pst) HIS4::LEU2-(NBam) ndj1::KanMx nup2(89-130)-GFP::CaURA3*	SK1	Table 1 (sporulation, *ndj1* background)	
	MATalpha ho::hisG leu2::hisG ura3(ΔSma-Pst) his4-X::LEU2-(NBam)-URA3 ndj1::KanMx nup2(89-130)-GFP::CaURA3			
SBY6414	*MATa ho::hisG leu2::hisG ura3(ΔSma-Pst) HIS4::LEU2-(NBam) ndj1::KanMx nup2(89-175)-GFP::CaURA3*	SK1	Table 1 (sporulation, *ndj1* background)	
	MATalpha ho::hisG leu2::hisG ura3(ΔSma-Pst) his4-X::LEU2-(NBam)-URA3 ndj1::KanMx nup2(89-175)-GFP::CaURA3			
SBY3981 x SBY6114	*MATalpha ho::hisG leu2::hisG ura3(ΔSma-Pst) his4-X::LEU2-(NBam)-URA3 nup2:KanMx ndj1::KanMx*	SK1	Table 1 (hemizygote sporulation in *ndj1* background)	
	MATa ho::hisG leu2::hisG ura3(ΔSma-Pst) HIS4::LEU2-(NBam) nup2(51-175)^{K101E}*-GFP::CaURA3 ndj1::KanMx*			
SBY3981 x SBY6081	*MATalpha ho::hisG leu2::hisG ura3(ΔSma-Pst) his4-X::LEU2-(NBam)-URA3 nup2:KanMx ndj1::KanMx*	SK1	Table 1 (hemizygote sporulation in *ndj1* background)	
	MATa ho::hisG leu2::hisG ura3(ΔSma-Pst) HIS4::LEU2-(NBam) nup2(51-175)^{F120Y}*-GFP::CaURA3 ndj1::KanMx*			
SBY3981 x SBY6264	*MATalpha ho::hisG leu2::hisG ura3(ΔSma-Pst) his4-X::LEU2-(NBam)-URA3 nup2:KanMx ndj1::KanMx*	SK1	Table 1 (hemizygote sporulation in *ndj1* background)	
	MATa ho::hisG leu2::hisG ura3(ΔSma-Pst) HIS4::LEU2-(NBam) nup2(89-130)-GFP::CaURA3 ndj1::KanMx			
SBY3981 x SBY6413	*MATalpha ho::hisG leu2::hisG ura3(ΔSma-Pst) his4-X::LEU2-(NBam)-URA3 nup2:KanMx ndj1::KanMx*	SK1	Table 1 (hemizygote sporulation in *ndj1* background)	
	MATa ho::hisG leu2::hisG ura3(ΔSma-Pst) HIS4::LEU2-(NBam) nup2(89-175)-GFP::CaURA3 ndj1::KanMx			

SBY6068	*MATa ho::hisG leu2::hisG ura3(ΔSma-Pst) HIS4::LEU2-(NBam) nup2(51-175)-GFP::CaURA3*<br>*MATalpha ho::hisG leu2::hisG ura3(ΔSma-Pst) his4-X::LEU2-(NBam)-URA3 nup2(51-175)-GFP::CaURA3*	SK1	Table 1, Figure 1D (sporulation, localization)	
SBY6068*	*MATa ho::hisG leu2::hisG ura3(ΔSma-Pst) HIS4::LEU2-(NBam) nup2(51-175)*-GFP::CaURA3*<br>*MATalpha ho::hisG leu2::hisG ura3(ΔSma-Pst) his4-X::LEU2-(NBam)-URA3 nup2(51-175)*-GFP::CaURA3*	SK1	Table 1, Figure 1D (sporulation, localization) point mutant versions of SBY6068	
SBY6074	*MATa ho::hisG leu2::hisG ura3(ΔSma-Pst) HIS4::LEU2-(NBam) nup2(Δ1-720)-GFP::CaURA3*<br>*MATalpha ho::hisG leu2::hisG ura3(ΔSma-Pst) his4-X::LEU2-(NBam)-URA3 nup2(Δ1-720)-GFP::CaURA3*	SK1	Table 1, Figure 1D (sporulation, localization)	
SBY6071	*MATa ho::hisG leu2::hisG ura3(ΔSma-Pst) HIS4::LEU2-(NBam) nup2(71-130)-GFP::CaURA3*<br>*MATalpha ho::hisG leu2::hisG ura3(ΔSma-Pst) his4-X::LEU2-(NBam)-URA3 nup2(71-130)-GFP::CaURA3*	SK1	Table 1, Figure 1D (sporulation, localization)	
SBY6263	*MATa ho::hisG leu2::hisG ura3(ΔSma-Pst) HIS4::LEU2-(NBam) nup2(89-130)-GFP::CaURA3*<br>*MATalpha ho::hisG leu2::hisG ura3(ΔSma-Pst) his4-X::LEU2-(NBam)-URA3 nup2(89-130)-GFP::CaURA3*	SK1	Table 1, Figure 1D (sporulation, localization)	
SBY3943<br>x<br>SBY6111	*MATalpha ho::hisG leu2::hisG ura3(ΔSma-Pst) his4-X::LEU2-(NBam)-URA3 nup2::KanMx*<br>*MATa ho::hisG leu2::hisG ura3(ΔSma-Pst) HIS4::LEU2-(NBam) nup2(51-175)*^{K101E}*-GFP::CaURA3*	SK1	Table1, Figure S1 (hemizygote sporulation in *NDJ1* background, localization)	
SBY3983<br>x<br>SBY6084	*MATalpha ho::hisG leu2::hisG ura3(ΔSma-Pst) his4-X::LEU2-(NBam)-URA3 nup2::KanMx*	SK1	Table1, Figure S1 (hemizygote sporulation in	

	MATa ho::hisG leu2::hisG ura3(ΔSma-Pst) HIS4::LEU2-(NBam) nup2(51-175)^{F120Y}-GFP::CaURA3		*NDJ1* background, localization)	
SBY3983<br>x<br>SBY6261	*MATalpha ho::hisG leu2::hisG ura3(ΔSma-Pst) his4-X::LEU2-(NBam)-URA3 nup2::KanMx*<br>*MATa ho::hisG leu2::hisG ura3(ΔSma-Pst) HIS4::LEU2-(NBam) nup2(89-130)-GFP::CaURA3*	SK1	Table1, Figure S1 (hemizygote sporulation in *NDJ1* background, localization)	
SBY3983<br>x<br>SBY6535	*MATalpha ho::hisG leu2::hisG ura3(ΔSma-Pst) his4-X::LEU2-(NBam)-URA3 nup2::KanMx*<br>*MATa ho::hisG leu2::hisG ura3(ΔSma-Pst) HIS4::LEU2-(NBam) nup2(89-175)-GFP::CaURA3*	SK1	Table1, Figure S1 (hemizygote sporulation in *NDJ1* background, localization)	
AH109	*MATa, trp1–901, leu2–3, 112, ura3–52, his3–200, gal4Δ, gal80Δ, LYS2:: GAL1UAS-GAL1TATA-HIS3, GAL2UAS-GAL2TATA-ADE2, URA3:: MEL1UAS-MEL1TATA-lacZ.*		Figures 2, S2 (Y2H)	Clontech
SBY6054	*MATa ho::hisG leu2::hisG ura3(ΔSma-Pst) HIS4::LEU2-(NBam) nup2(51-175)-GFP::CaURA3 ndt80::Hph*<br>*MATalpha ho::hisG leu2::hisG ura3(ΔSma-Pst) his4-X::LEU2-(NBam)-URA3 nup2(51-175)-GFP::CaURA3 ndt80::Hph*	SK1	Figures 3A, B (chromosome spreads) wt control	
SBY6119	*MATa ho::hisG leu2::hisG ura3(ΔSma-Pst) HIS4::LEU2-(NBam) nup2(51-175)^{K101E}-GFP::CaURA3 ndt80::Hph*<br>*MATalpha ho::hisG leu2::hisG ura3(ΔSma-Pst) his4-X::LEU2-(NBam)-URA3 nup2(51-175)^{K101E}-GFP::CaURA3 ndt80::Hph*	SK1	Figure 3A (chromosome spreads) K101E version of SBY6054	
SBY6107	*MATa ho::hisG leu2::hisG ura3(ΔSma-Pst) HIS4::LEU2-(NBam) nup2(51-175)^{F120Y}-GFP::CaURA3 ndt80::Hph*<br>*MATalpha ho::hisG leu2::hisG ura3(ΔSma-Pst) his4-X::LEU2-(NBam)-URA3 nup2(51-175)^{F120Y}-GFP::CaURA3 ndt80::Hph*	SK1	Figure 3A (chromosome spreads) F120Y version of SBY6054	

SBY6496	*MATa ho::hisG leu2::hisG ura3(ΔSma-Pst) HIS4::LEU2-(NBam) nup2(51-175)^{N97S}-GFP::CaURA3 ndt80::Hph*	SK1	Figure 3A (chromosome spreads) N97S version of SBY6054	
	MATalpha ho::hisG leu2::hisG ura3(ΔSma-Pst) his4-X::LEU2-(NBam)-URA3 nup2(51-175)^{N97S}-GFP::CaURA3 ndt80::Hph			
SBY6499	*MATa ho::hisG leu2::hisG ura3(ΔSma-Pst) HIS4::LEU2-(NBam) nup2(51-175)^{V108D}-GFP::CaURA3 ndt80::Hph*	SK1	Figure 3A chromosome spreads (V108D version of SBY6054)	
	MATalpha ho::hisG leu2::hisG ura3(ΔSma-Pst) his4-X::LEU2-(NBam)-URA3 nup2(51-175)^{V108D}-GFP::CaURA3 ndt80::Hph			
SBY6502	*MATa ho::hisG leu2::hisG ura3(ΔSma-Pst) HIS4::LEU2-(NBam) nup2(51-175)^{R117W}-GFP::CaURA3 ndt80::Hph*	SK1	Figure 3A chromosome spreads (R117W version of SBY6054)	
	MATalpha ho::hisG leu2::hisG ura3(ΔSma-Pst) his4-X::LEU2-(NBam)-URA3 nup2(51-175)^{R117W}-GFP::CaURA3 ndt80::Hph			
SBY6490	*MATa ho::hisG leu2::hisG ura3(ΔSma-Pst) HIS4::LEU2-(NBam) nup2(51-175)^{L93P}-GFP::CaURA3 ndt80::Hph*	SK1	Figure 3A chromosome spreads (L93P version of SBY6054)	
	MATalpha ho::hisG leu2::hisG ura3(ΔSma-Pst) his4-X::LEU2-(NBam)-URA3 nup2(51-175)^{L93P}-GFP::CaURA3 ndt80::Hph			
SBY6493	*MATa ho::hisG leu2::hisG ura3(ΔSma-Pst) HIS4::LEU2-(NBam) nup2(51-175)^{A102P}-GFP::CaURA3 ndt80::Hph*	SK1	Figure 3A chromosome spreads (A102P version of SBY6054)	
	MATalpha ho::hisG leu2::hisG ura3(ΔSma-Pst) his4-X::LEU2-(NBam)-URA3 nup2(51-175)^{A102P}-GFP::CaURA3 ndt80::Hph			
SBY6101	*MATa ho::hisG leu2::hisG ura3(ΔSma-Pst) HIS4::LEU2-(NBam) nup2(Δ1-720)-GFP::CaURA3 ndt80::Hph*	SK1	Figures 3A, B chromosome spreads (GFP control)	
	MATalpha ho::hisG leu2::hisG ura3(ΔSma-Pst) his4-X::LEU2-			

	(NBam)-URA3 nup2(Δ1-720)-GFP::CaURA3 ndt80::Hph			
SBY6269	*MATa ho::hisG leu2::hisG ura3(ΔSma-Pst) HIS4::LEU2-(NBam) nup2(71-130)-GFP::CaURA3 ndt80::Hph*<br>*MATalpha ho::hisG leu2::hisG ura3(ΔSma-Pst) his4-X::LEU2-(NBam)-URA3 nup2(71-130)-GFP::CaURA3 ndt80::Hph*	SK1	Figure 3A chromosome spreads (mar(71-130) version of SBY6054)	
SBY6272	*MATa ho::hisG leu2::hisG ura3(ΔSma-Pst) HIS4::LEU2-(NBam) nup2(89-130)-GFP::CaURA3 ndt80::Hph*<br>*MATalpha ho::hisG leu2::hisG ura3(ΔSma-Pst) his4-X::LEU2-(NBam)-URA3 nup2(89-130)-GFP::CaURA3 ndt80::Hph*	SK1	Figure 3A chromosome spreads (mar(89-130) version of SBY6054)	
SBY6278	*MATa ho::hisG leu2::hisG ura3(ΔSma-Pst) HIS4::LEU2-(NBam) nup2(51-175)-GFP::CaURA3 ndt80::Hph nup60::KanMx*<br>*MATalpha ho::hisG leu2::hisG ura3(ΔSma-Pst) his4-X::LEU2-(NBam)-URA3 nup2(51-175)-GFP::CaURA3 ndt80::Hph nup60::KanMx*	SK1	Figure 3B chromosome spreads (*nup60Δ* version of SBY6054)	
SBY5217	*MATa ho::hisG leu2::hisG ura3(ΔSma-Pst) HIS4::LEU2-(NBam) nup60::Hph*<br>*MATalpha ho::hisG leu2::hisG ura3(ΔSma-Pst) his4-X::LEU2-(NBam)-URA3 nup60::Hph*	SK1	Figures 4, S4 (growth, sporulation, HU sensitivity)	(Chu *et al.* 2017)
SBY6420	*MATa ho::hisG leu2::hisG ura3(ΔSma-Pst) HIS4::LEU2-(NBam) nup60(189-388)*<br>*MATalpha ho::hisG leu2::hisG ura3(ΔSma-Pst) his4-X::LEU2-(NBam)-URA3 nup60(189-388)*	SK1	Figures 4, S4, 5 (growth, sporulation, HU sensitivity, meiotic time course)	
SBY6423	*MATa ho::hisG leu2::hisG ura3(ΔSma-Pst) HIS4::LEU2-(NBam) nup60(1-388)*<br>*MATalpha ho::hisG leu2::hisG ura3(ΔSma-Pst) his4-X::LEU2-(NBam)-URA3 nup60(1-388)*	SK1	Figures 4, S4 (growth, sporulation, HU sensitivity)	
SBY6426	*MATa ho::hisG leu2::hisG ura3(ΔSma-Pst) HIS4::LEU2-(NBam) nup60(189-539)*	SK1	Figures 4, S4 (growth,	

	MATalpha ho::hisG leu2::hisG ura3(ΔSma-Pst) his4-X::LEU2-(NBam)-URA3 nup60(189-539)		sporulation, HU sensitivity)	
SBY6429	*MATa ho::hisG leu2::hisG ura3(ΔSma-Pst) HIS4::LEU2-(NBam) nup60(Δ189-388)*	SK1	Figures 4, S4 (growth, sporulation, HU sensitivity)	
	MATalpha ho::hisG leu2::hisG ura3(ΔSma-Pst) his4-X::LEU2-(NBam)-URA3 nup60(Δ189-388)			
SBY1904	*MATa ho::hisG leu2::hisG ura3(ΔSma-Pst) HIS4::LEU2-(NBam) ndj1::KanMx*	SK1	Figure 4 (sporulation)	(Chu *et al.* 2017)
	MATalpha ho::hisG leu2::hisG ura3(ΔSma-Pst) his4-X::LEU2-(NBam)-URA3 ndj1::KanMx			
SBY5222	*MATa ho::hisG leu2::hisG ura3(ΔSma-Pst) HIS4::LEU2-(NBam) ndj1::KanMx nup60::Hph*	SK1	Figure 4 (sporulation)	
	MATalpha ho::hisG leu2::hisG ura3(ΔSma-Pst) his4-X::LEU2-(NBam)-URA3 ndj1::KanMx nup60::Hph			
SBY6293	*MATa ho::hisG leu2::hisG ura3(ΔSma-Pst) HIS4::LEU2-(NBam) ndj1::KanMx nup60(189-388)*	SK1	Figures 4, 5 (sporulation, meiotic time courses)	
	MATalpha ho::hisG leu2::hisG ura3(ΔSma-Pst) his4-X::LEU2-(NBam)-URA3 ndj1::KanMx nup60(189-388)			
SBY6296	*MATa ho::hisG leu2::hisG ura3(ΔSma-Pst) HIS4::LEU2-(NBam) ndj1::KanMx nup60(1-388)*	SK1	Figure 4 (sporulation)	
	MATalpha ho::hisG leu2::hisG ura3(ΔSma-Pst) his4-X::LEU2-(NBam)-URA3 ndj1::KanMx nup60(1-388)			
SBY6299	*MATa ho::hisG leu2::hisG ura3(ΔSma-Pst) HIS4::LEU2-(NBam) ndj1::KanMx nup60(189-539)*	SK1	Figure 4 (sporulation)	
	MATalpha ho::hisG leu2::hisG ura3(ΔSma-Pst) his4-X::LEU2-(NBam)-URA3 ndj1::KanMx nup60(189-539)			
SBY6302	*MATa ho::hisG leu2::hisG ura3(ΔSma-Pst) HIS4::LEU2-(NBam) ndj1::KanMx nup60(Δ189-388)*	SK1	Figure 4 (sporulation)	

	MATalpha ho::hisG leu2::hisG ura3(ΔSma-Pst) his4-X::LEU2-(NBam)-URA3 ndj1::KanMx nup60(Δ189-388)			
SBY6304	*MATalpha ho::hisG leu2::hisG ura3(ΔSma-Pst) his4-X::LEU2-(NBam)-URA3 NUP60-GFP::KanMx*	SK1	Figure S5A (GFP fusion growth)	
SBY6307	*MATalpha ho::hisG leu2::hisG ura3(ΔSma-Pst) his4-X::LEU2-(NBam)-URA3 nup60(Δ1-539)-GFP::KanMx*	SK1	Figure S5A (GFP fusion growth)	
SBY6310	*MATalpha ho::hisG leu2::hisG ura3(ΔSma-Pst) his4-X::LEU2-(NBam)-URA3 nup60(189-388)-GFP::KanMx*	SK1	Figure S5A (GFP fusion growth)	
SBY6313	*MATa ho::hisG leu2::hisG MATalpha ho::hisG leu2::hisG ura3(ΔSma-Pst) his4-X::LEU2-(NBam)-URA3 nup60(1-388)-GFP::KanMx*	SK1	Figure S5A (GFP fusion growth)	
SBY6316	*MATalpha ho::hisG leu2::hisG ura3(ΔSma-Pst) his4-X::LEU2-(NBam)-URA3 nup60(189-539)-GFP::KanMx*	SK1	Figure S5A (GFP fusion growth)	

SBY6382	*MATalpha ho::hisG leu2::hisG ura3(ΔSma-Pst) his4-X::LEU2-(NBam)-URA3 nup60(Δ189-388)-GFP::KanMx*	SK1	Figure S5A (GFP fusion growth)	
SBY6320	*MATa ho::hisG leu2::hisG ura3(ΔSma-Pst) HIS4::LEU2-(NBam) ndj1::KanMx NUP60-GFP::KanMx*	SK1	Figure S5B (GFP fusion sporulation)	
	MATalpha ho::hisG leu2::hisG ura3(ΔSma-Pst) his4-X::LEU2-(NBam)-URA3 ndj1::KanMx NUP60-GFP::KanMx			
SBY6323	*MATa ho::hisG leu2::hisG ura3(ΔSma-Pst) HIS4::LEU2-(NBam) ndj1::KanMx nup60(Δ1-539)-GFP::KanMx*	SK1	Figure S5B (GFP fusion sporulation)	
	MATalpha ho::hisG leu2::hisG ura3(ΔSma-Pst) his4-X::LEU2-(NBam)-URA3 ndj1::KanMx nup60(Δ1-539)-GFP::KanMx			
SBY6326	*MATa ho::hisG leu2::hisG ura3(ΔSma-Pst) HIS4::LEU2-(NBam) ndj1::KanMx nup60(189-388)-GFP::KanMx*	SK1	Figure S5B (GFP fusion sporulation)	
	MATalpha ho::hisG leu2::hisG ura3(ΔSma-Pst) his4-X::LEU2-(NBam)-URA3 ndj1::KanMx nup60(189-388)-GFP::KanMx			
SBY6329	*MATa ho::hisG leu2::hisG ura3(ΔSma-Pst) HIS4::LEU2-(NBam) ndj1::KanMx nup60(1-388)-GFP::KanMx*	SK1	Figure S5B (GFP fusion sporulation)	
	MATalpha ho::hisG leu2::hisG ura3(ΔSma-Pst) his4-X::LEU2-(NBam)-URA3 ndj1::KanMx nup60(1-388)-GFP::KanMx			
SBY6332	*MATa ho::hisG leu2::hisG ura3(ΔSma-Pst) HIS4::LEU2-(NBam) ndj1::KanMx nup60(189-539)-GFP::KanMx*	SK1	Figure S5B (GFP fusion sporulation)	
	MATalpha ho::hisG leu2::hisG ura3(ΔSma-Pst) his4-X::LEU2-(NBam)-URA3 ndj1::KanMx nup60(189-539)-GFP::KanMx			
SBY6335	*MATa ho::hisG leu2::hisG ura3(ΔSma-Pst) HIS4::LEU2-(NBam) ndj1::KanMx nup60(Δ189-388)-GFP::KanMx*	SK1	Figure S5B (GFP fusion sporulation)	

	MATalpha ho::hisG leu2::hisG ura3(ΔSma-Pst) his4-X::LEU2-(NBam)-URA3 ndj1::KanMx nup60(Δ189-388)-GFP::KanMx			
SBY3945	*MATa ho::hisG leu2::hisG ura3(ΔSma-Pst) HIS4::LEU2-(NBam) nup2::KanMx*	SK1	Figure 5 (Meiotic time courses)	(Chu *et al.* 2017)
	MATalpha ho::hisG leu2::hisG ura3(ΔSma-Pst) his4-X::LEU2-(NBam)-URA3 nup2::KanMx			
SBY6432	*MATa ho::hisG leu2::hisG ura3(ΔSma-Pst) HIS4::LEU2-(NBam) nup2::KanMx nup60(189-388)*	SK1	Figure 5, Table 2 (Meiotic time courses, *nup60* epistasis)	
	MATalpha ho::hisG leu2::hisG ura3(ΔSma-Pst) his4-X::LEU2-(NBam)-URA3 nup2::KanMx nup60(189-388)			
SBY3983	*MATa ho::hisG leu2::hisG ura3(ΔSma-Pst) HIS4::LEU2-(NBam) nup2::KanMx ndj1::KanMx*	SK1	Figure 5, Table 2 (Meiotic time courses, *nup60* epistasis)	(Chu *et al.* 2017)
	MATalpha ho::hisG leu2::hisG ura3(ΔSma-Pst) his4-X::LEU2-(NBam)-URA3 nup2::KanMx ndj1::KanMx			
SBY6296	*MATa ho::hisG leu2::hisG ura3(ΔSma-Pst) HIS4::LEU2-(NBam) ndj1::KanMx nup60(189-388)*	SK1	Figure 5, Table 2 (Meiotic time courses, *nup60* epistasis)	
	MATalpha ho::hisG leu2::hisG ura3(ΔSma-Pst) his4-X::LEU2-(NBam)-URA3 ndj1::KanMx nup60(189-388)			
SBY6254	*MATa ho::hisG leu2::hisG ura3(ΔSma-Pst) HIS4::LEU2-(NBam) NUP60-GFP::KanMx nup2(51-175)-mCherry::Nat ndt80::Hph*	SK1	Figures 6 and 7 (NUP60-GFP localization and spreads)	
	MATalpha ho::hisG leu2::hisG ura3(ΔSma-Pst) his4-X::LEU2-(NBam)-URA3 NUP60-GFP::KanMx nup2(51-175)-mCherry::Nat ndt80::Hph			
SBY6338	*MATa ho::hisG leu2::hisG ura3(ΔSma-Pst) HIS4::LEU2-(NBam) nup60(Δ1-539)-GFP::KanMx nup2(51-175)-mCherry::Nat ndt80::Hph*	SK1	Figures 6 and 7 (NUP60-GFP localization and spreads)	

	MATalpha ho::hisG leu2::hisG ura3(ΔSma-Pst) his4-X::LEU2-(NBam)-URA3 nup60(Δ1-539)-GFP::KanMx nup2(51-175)-mCherry::Nat ndt80::Hph			
SBY6344	*MATa ho::hisG leu2::hisG ura3(ΔSma-Pst) HIS4::LEU2-(NBam) nup60(1-388)-GFP::KanMx nup2(51-175)-mCherry::Nat ndt80::Hph*<br>*MATalpha ho::hisG leu2::hisG ura3(ΔSma-Pst) his4-X::LEU2-(NBam)-URA3 nup60(1-388)-GFP::KanMx nup2(51-175)-mCherry::Nat ndt80::Hph*	SK1	Figures 6 and 7 (NUP60-GFP localization and spreads)	
SBY6341	*MATa ho::hisG leu2::hisG ura3(ΔSma-Pst) HIS4::LEU2-(NBam) nup60(189-388)-GFP::KanMx nup2(51-175)-mCherry::Nat ndt80::Hph*<br>*MATalpha ho::hisG leu2::hisG ura3(ΔSma-Pst) his4-X::LEU2-(NBam)-URA3 nup60(189-388)-GFP::KanMx nup2(51-175)-mCherry::Nat ndt80::Hph*	SK1	Figures 6 and 7 (NUP60-GFP localization and spreads)	
SBY6347	*MATa ho::hisG leu2::hisG ura3(ΔSma-Pst) HIS4::LEU2-(NBam) nup60(189-539)-GFP::KanMx nup2(51-175)-mCherry::Nat ndt80::Hph*<br>*MATalpha ho::hisG leu2::hisG ura3(ΔSma-Pst) his4-X::LEU2-(NBam)-URA3 nup60(189-539)-GFP::KanMx nup2(51-175)-mCherry::Nat ndt80::Hph*	SK1	Figures 6 and 7 (NUP60-GFP localization and spreads)	
SBY6350	*MATa ho::hisG leu2::hisG ura3(ΔSma-Pst) HIS4::LEU2-(NBam) nup60(Δ189-388)-GFP::KanMx nup2(51-175)-mCherry::Nat ndt80::Hph*<br>*MATalpha ho::hisG leu2::hisG ura3(ΔSma-Pst) his4-X::LEU2-(NBam)-URA3 nup60(Δ189-388)-GFP::KanMx nup2(51-175)-mCherry::Nat ndt80::Hph*	SK1	Figure 6 (NUP60-GFP localization)	
SBY4959	*MATa ho::hisG leu2::hisG ura3(DSma-Pst) HIS4::LEU2-(NBam) ndj1::KanMx nup2::Nat*<br>*MATalpha ho::hisG leu2::hisG ura3(DSma-Pst) his4-X::LEU2-*	SK1	Table 3 (Suppression of *nup2 ndj1*)	

	(NBam)-URA3 ndj1::KanMx nup2::Nat			
SBY6365	*MATa ho::hisG leu2::hisG ura3(ΔSma-Pst) HIS4::LEU2-(NBam) ndj1::KanMx nup2::Nat NUP60-GFP::KanMx*<br>*MATalpha ho::hisG leu2::hisG ura3(ΔSma-Pst) his4-X::LEU2-(NBam)-URA3 ndj1::KanMx nup2::Nat NUP60-GFP::KanMx*	SK1	Table 3 (Suppression of *nup2 ndj1*)	
SBY6371	*MATa ho::hisG leu2::hisG ura3(ΔSma-Pst) HIS4::LEU2-(NBam) ndj1::KanMx nup2::Nat nup60(1-388)-GFP::KanMx*<br>*MATalpha ho::hisG leu2::hisG ura3(ΔSma-Pst) his4-X::LEU2-(NBam)-URA3 ndj1::KanMx nup2::Nat nup60(1-388)-GFP::KanMx*	SK1	Table 3 (Suppression of *nup2 ndj1*)	
SBY6356	*MATa ho::hisG leu2::hisG ura3(ΔSma-Pst) HIS4::LEU2-(NBam) ndj1::KanMx nup2::Nat nup60(1-388)*<br>*MATalpha ho::hisG leu2::hisG ura3(ΔSma-Pst) his4-X::LEU2-(NBam)-URA3 ndj1::KanMx nup2::Nat nup60(1-388)*	SK1	Table 3 (Suppression of *nup2 ndj1*)	
SBY6436	*MATa ho::hisG leu2::hisG ura3(ΔSma-Pst) HIS4::LEU2-(NBam) ndj1::KanMx nup2::Nat NUP60-mCherry::KanMx*<br>*MATalpha ho::hisG leu2::hisG ura3(ΔSma-Pst) his4-X::LEU2-(NBam)-URA3 ndj1::KanMx nup2::Nat NUP60-mCherry::KanMx*	SK1	Table 3 (Suppression of *nup2 ndj1*)	
SBY6439	*MATa ho::hisG leu2::hisG ura3(ΔSma-Pst) HIS4::LEU2-(NBam) ndj1::KanMx nup2::Nat NUP60-13myc::KanMx*<br>*MATalpha ho::hisG leu2::hisG ura3(ΔSma-Pst) his4-X::LEU2-(NBam)-URA3 ndj1::KanMx nup2::Nat NUP60-13myc::KanMx*	SK1	Table 3 (Suppression of *nup2 ndj1*)	

SBY3982 x SBY6364	*MATa ho::hisG leu2::hisG ura3(ΔSma-Pst) HIS4::LEU2-(NBam) ndj1::KanMx nup2::Nat*	SK1	Table 3 (Suppression of *nup2 ndj1*)	
	MATalpha ho::hisG leu2::hisG ura3(ΔSma-Pst) his4-X::LEU2-(NBam)-URA3 ndj1::KanMx nup2::Nat NUP60-GFP::KanMxB			
SBY3982 x SBY6435	*MATa ho::hisG leu2::hisG ura3(ΔSma-Pst) HIS4::LEU2-(NBam) ndj1::KanMx nup2::Nat*	SK1	Table 3 (Suppression of *nup2 ndj1*)	
	MATalpha ho::hisG leu2::hisG ura3(ΔSma-Pst) his4-X::LEU2-(NBam)-URA3 ndj1::KanMx nup2::Nat NUP60-mCherry::KanMx			
SBY3982 x SBY6370	*MATa ho::hisG leu2::hisG ura3(ΔSma-Pst) HIS4::LEU2-(NBam) ndj1::KanMx nup2::Nat*	SK1	Table 3 (Suppression of *nup2 ndj1*)	
	MATalpha ho::hisG leu2::hisG ura3(ΔSma-Pst) his4-X::LEU2-(NBam)-URA3 ndj1::KanMx nup2::Nat nup60(1-388)-GFP::KanMx			
SBY3982 x SBY6355	*MATa ho::hisG leu2::hisG ura3(ΔSma-Pst) HIS4::LEU2-(NBam) ndj1::KanMx nup2::Nat*	SK1	Table 3 (Suppression of *nup2 ndj1*)	
	MATalpha ho::hisG leu2::hisG ura3(ΔSma-Pst) his4-X::LEU2-(NBam)-URA3 ndj1::KanMx nup2::Nat nup60(1-388)			
SBY6484	*MATa ho::hisG leu2::hisG ura3(ΔSma-Pst) HIS4::LEU2-(NBam) NUP60-GFP::KanMx ndt80::Hph*	SK1	Figure 8 (Nup60-GFP binding with and without Nup2)	
	MATalpha ho::hisG leu2::hisG ura3(ΔSma-Pst) his4-X::LEU2-(NBam)-URA3 NUP60-GFP::KanMx ndt80::Hph			
SBY6487	*MATa ho::hisG leu2::hisG ura3(ΔSma-Pst) HIS4::LEU2-(NBam) NUP60-GFP::KanMx nup2::NatMx ndt80::Hph*	SK1	Figure 8 (Nup60-GFP binding with and without Nup2)	
	MATalpha ho::hisG leu2::hisG ura3(ΔSma-Pst) his4-X::LEU2-(NBam)-URA3 NUP60-GFP::KanMx nup2::NatMx ndt80::Hph			

SBY6505	*MATa ho::hisG leu2::hisG ura3(ΔSma-Pst) HIS4::LEU2-(NBam) nup60(189-388)-GFP::KanMx ndt80::Hph*	SK1	Figure 8 (Nup60-GFP binding with and without Nup2)	
	MATalpha ho::hisG leu2::hisG ura3(ΔSma-Pst) his4-X::LEU2-(NBam)-URA3 nup60(189-388)-GFP::KanMx ndt80::Hph			
SBY6442	*MATa ho::hisG leu2::hisG ura3(ΔSma-Pst) HIS4::LEU2-(NBam) csm4::Hph*	SK1	Table 2 (*nup60* epistasis)	
	MATalpha ho::hisG leu2::hisG ura3(ΔSma-Pst) his4-X::LEU2-(NBam)-URA3 csm4::Hph			
SBY6448	*MATa ho::hisG leu2::hisG ura3(ΔSma-Pst) HIS4::LEU2-(NBam) nup60(189-388) csm4::Hph*	SK1	Table 2 (*nup60* epistasis)	
	MATalpha ho::hisG leu2::hisG ura3(ΔSma-Pst) his4-X::LEU2-(NBam)-URA3 nup60(189-388) csm4::Hph			
SBY6445	*MATa ho::hisG leu2::hisG ura3(ΔSma-Pst) HIS4::LEU2-(NBam) nup60::Hph csm4::Hph*	SK1	Table 2 (*nup60* epistasis)	
	MATalpha ho::hisG leu2::hisG ura3(ΔSma-Pst) his4-X::LEU2-(NBam)-URA3 nup60::Hph csm4::Hph			
SBY6451	*MATa ho::hisG leu2::hisG ura3(ΔSma-Pst) HIS4::LEU2-(NBam) nup2::NatMx csm4::Hph*	SK1	Table 2 (*nup60* epistasis)	
	MATalpha ho::hisG leu2::hisG ura3(ΔSma-Pst) his4-X::LEU2-(NBam)-URA3 nup2::NatMx csm4::Hph			
SBY6353	*MATa ho::hisG leu2::hisG ura3(ΔSma-Pst) HIS4::LEU2-(NBam) nup60(189-388) nup2::KanMx ndj1::KanMx*	SK1	Table 2 (nup60 epistasis)	
	MATalpha ho::hisG leu2::hisG ura3(ΔSma-Pst) his4-X::LEU2-(NBam)-URA3 nup60(189-388) nup2::KanMx ndj1::KanMx			
SBY6457	*MATa ho::hisG leu2::hisG ura3(ΔSma-Pst) HIS4::LEU2-(NBam) 3HA-NUP60*	SK1	Figure S8A (HA-Nup60 sporulation)	
	MATalpha ho::hisG leu2::hisG ura3(ΔSma-Pst) his4-X::LEU2-(NBam)-URA3 3HA-NUP60			

SBY6460	*MATa ho::hisG leu2::hisG ura3(ΔSma-Pst) HIS4::LEU2-(NBam) 3HA-NUP60 ndj1::Hph*<br>*MATalpha ho::hisG leu2::hisG ura3(ΔSma-Pst) his4-X::LEU2-(NBam)-URA3 3HA-NUP60 ndj1::Hph*	SK1	Figure S8A (HA-Nup60 sporulation)	
SBY6463	*MATa ho::hisG leu2::hisG ura3(ΔSma-Pst) HIS4::LEU2-(NBam) 3HA-NUP60 nup2::NatMx*<br>*MATalpha ho::hisG leu2::hisG ura3(ΔSma-Pst) his4-X::LEU2-(NBam)-URA3 3HA-NUP60 nup2::NatMx*	SK1	Figure S8A (HA-Nup60 sporulation)	
SBY6466	*MATa ho::hisG leu2::hisG ura3(ΔSma-Pst) HIS4::LEU2-(NBam) 3HA-NUP60 ndj1::Hph nup2::NatMx*<br>*MATalpha ho::hisG leu2::hisG ura3(ΔSma-Pst) his4-X::LEU2-(NBam)-URA3 3HA-NUP60 ndj1::Hph nup2::NatMx*	SK1	Figure S8A (HA-Nup60 sporulation)	
SBY6469	*MATa ho::hisG leu2::hisG ura3(ΔSma-Pst) HIS4::LEU2-(NBam) NUP60-3HA(Δ1F)::KanMx*<br>*MATalpha ho::hisG leu2::hisG ura3(ΔSma-Pst) his4-X::LEU2-(NBam)-URA3 NUP60-3HA(Δ1F)::KanMx*	SK1	Figure S8B (Nup60-HA western)	
SBY6472	*MATa ho::hisG leu2::hisG ura3(ΔSma-Pst) HIS4::LEU2-(NBam) NUP60-3HA(Δ1F)::KanMx ndj1::Hph*<br>*MATalpha ho::hisG leu2::hisG ura3(ΔSma-Pst) his4-X::LEU2-(NBam)-URA3 NUP60-3HA(Δ1F)::KanMx ndj1::Hph*	SK1	Figure S8B (Nup60-HA western)	
SBY6475	*MATa ho::hisG leu2::hisG ura3(ΔSma-Pst) HIS4::LEU2-(NBam) NUP60-3HA(Δ1F)::KanMx nup2::NatMx*<br>*MATalpha ho::hisG leu2::hisG ura3(ΔSma-Pst) his4-X::LEU2-(NBam)-URA3 NUP60-3HA(Δ1F)::KanMx nup2::NatMx*	SK1	Figure S8B (Nup60-HA western)	
SBY6478	*MATa ho::hisG leu2::hisG ura3(ΔSma-Pst) HIS4::LEU2-(NBam) NUP60-*	SK1	Figure S8B (Nup60-HA western)	

	3HA(Δ1F)::KanMx nup2::NatMx ndj1::Hph			
	MATalpha ho::hisG leu2::hisG ura3(ΔSma-Pst) his4-X::LEU2-(NBam)-URA3 NUP60-3HA(Δ1F)::KanMx nup2::NatMx ndj1::Hph			
SBY6481	*MATa ho::hisG leu2::hisG ura3(ΔSma-Pst) HIS4::LEU2-(NBam) NUP60-mCherry::KanMx nup2(51-175)-GFP::CaURA3 ndt80::Hph*	SK1	Figure S9A (Nup60-mCherry spreads)	
	MATalpha ho::hisG leu2::hisG ura3(ΔSma-Pst) his4-X::LEU2-(NBam)-URA3 NUP60-mCherry::KanMx nup2(51-175)-GFP::CaURA3 ndt80::Hph			
SBY6410	*MATa ho::hisG leu2::hisG ura3(ΔSma-Pst) HIS4::LEU2-(NBam) NUP60-3HA::KanMx nup2(51-175)-mCherry::NatMx ndt80::Hph*	SK1	Figure S9B (Nup60-3HA spreads)	
	MATalpha ho::hisG leu2::hisG ura3(ΔSma-Pst) his4-X::LEU2-(NBam)-URA3 NUP60-3HA::KanMx nup2(51-175)-mCherry::NatMx ndt80::Hph			

Table S2. Plasmids used in this study

Plasmid name	Description	Source/ Reference
pCR-BluntII-TOPO	cloning vector	Thermo Fisher/Invitrogen
pSB740	MAR-GFP::URA3 subcloned into pCR-Blunt II-TOPO	
pGBKT7	Gal4 DBD vector	Clontech
pACT2-2	Gal4 AD vector	(Arora *et al.* 2004)
pSB655	SK1 Nup2(51-175) coding sequence subcloned into pGBKT7	
pSB745	Nup2(51-175)^{F100S} coding sequence subcloned into pGBKT7	
pSB746	Nup2(51-175)^{K101E} coding sequence subcloned into pGBKT7	
pSB747	Nup2(51-175)^{R117W} coding sequence subcloned into pGBKT7	
pSB748	Nup2(51-175)^{L98P} coding sequence subcloned into pGBKT7	
pSB749	Nup2(51-175)^{A102P} coding sequence subcloned into pGBKT7	
pSB750	Nup2(51-175)^{L119P} coding sequence subcloned into pGBKT7	
pSB751	Nup2(51-175)^{Y123C} coding sequence subcloned into pGBKT7	
pSB752	Nup2(51-175)^{D115V} coding sequence subcloned into pGBKT7	
pSB753	Nup2(51-175)^{L93P} coding sequence subcloned into pGBKT7	
pSB754	Nup2(51-175)^{L96S} coding sequence subcloned into pGBKT7	
pSB755	Nup2(51-175)^{N97S} coding sequence subcloned into pGBKT7	
pSB756	Nup2(51-175)^{V108D} coding sequence subcloned into pGBKT7	
pSB757	Nup2(51-175)^{F120Y} coding sequence subcloned into pGBKT7	
pSB758	Nup2(51-175)^{Y126N} coding sequence subcloned into pGBKT7	
pSB759	Nup2(51-175)^{L116S} coding sequence subcloned into pGBKT7	
pSB743	Nup2(71-130) coding sequence subcloned into pGBKT7	
pSB744	Nup2(89-130) coding sequence subcloned into pGBKT7	
pSB741	SK1 Nup60 coding sequence subcloned into pACT2-2	
pSB742	SK1 Nup60(188-388) coding sequence subcloned into pACT2-2	

Table S3. Primers for PCR mutagenesis and Y2H plasmid construction

Primer name	Sequence	Description
oSB1274	GGGTTCGACTCCCCGTATC	forward primer for amplifying the full MAR-GFP::URA3 fragment
		MAR mutagenesis: forward primer for amplifying the 5' fragment containing the MAR
oSB1774	TGCGCGATGTTTTAACGAAG	reverse primer for amplifying the full MAR-GFP::URA3 fragment
		MAR mutagenesis: reverse primer for amplifying the 3' fragment containing GFP-URA3
oSB1918	TAAACCAGCACCGTCACCC	MAR mutagenesis: reverse primer for amplifying the 5' fragment containing MAR
oSB1919	GGGTGACGGTGCTGGTTTA	MAR mutagenesis: forward primer for amplifying the 3' fragment containing GFP-URA3
oSB1616	ACTGGAATTCATGAAACCTTTTGGTTCTGCAA	Y2H: forward primer for insertion of Nup2(51-175) coding sequence into pGBKT7
oSB1617	ACTGGGATCCTTACTTGGGTCCCTCCACCTTAACC	Y2H: reverse primer for insertion of Nup2(51-175) coding sequence into pGBKT7
oSB1920	ACTGCAACTAGTATGCATCGTAAATCATTGAGGAGGGCTAG	Y2H: forward primer for insertion of Nup60 coding sequence into pACT2-2
oSB1921	TAAGCACCATGGCAAAGGTATATAGGGACTTGAAAGCCTCAAC	Y2H: reverse primer for insertion of Nup60 coding sequence into pACT2-2
oSB1922	ACTGCAACTAGTATGCCGACCTTCAACCCAAAATATGATACTTCAAATG	Y2H: forward primer for insertion of Nup60(188-388)

		coding sequence into pACT2-2
oSB1923	TAAGCACCATGGCACCATCCTTCTTTTCAGGTGAGGTTTCA G	Y2H: reverse primer for insertion of Nup60(188-388) coding sequence into pACT2-2
oSB1924	ACTGGAATTCATGAACCGGGCGGACGGCACTG	Y2H: forward primer for insertion of Nup2(71-130) coding sequence into pGBKT7
oSB1925	ACTGGAATTCATGAGCAATTCCAGACTAAAAGCATTGAACC	Y2H: forward primer for insertion of Nup2(89-130) coding sequence into pGBKT7
oSB1926	ACTGGGATCCTTAGATATTCTTTATGTATAATTCGTACCTG GTG	Y2H: reverse primer for insertion of Nup2(71-130) and Nup2(89-130) coding sequences into pGBKT7

Supplemental methods

Western blot

Yeast extracts were prepared by alkaline lysis (von der Haar 2007) and resolved on a 4-15% Mini-PROTEAN TGX gel (BioRad, 4561085). Gel electrophoresis and wet transfer to PVDF were carried out according to standard procedures (Bolt and Mahoney 1997). Western blot detection was performed using ECL reagents and the manufacturer's instructions for PBS-based buffers (Amersham, RPN2108). Membranes were imaged with an ImageQuant LAS4000.

Antibodies

Primary antibodies used were mouse monoclonal antibody to HA (Santa Cruz Biotechnology SC-7392; RRID:AB_627809), rabbit polyclonal antibody to β-tubulin (Abcam AB15568; RRID:AB_2210952), chicken polyclonal antibody to GFP (Novus NB100-1614; RRID:AB_10001164), and rabbit polyclonal antibody to mCherry (Novus NBP2-25157; RRID:AB_2753204). The secondary antibodies used were goat anti-chicken 488 (Thermofisher A11039; RRID:AB_2534096), goat anti-rabbit 594 (ThermofisherA11012; RRID:AB_2534079), donkey anti-mouse 488 (Invitrogen A32766; RRID:AB_2762823), and the anti-rabbit and anti-mouse HRP-conjugated secondary antibodies provided in the ECL kit. All antibodies were used at a 1:1000 dilution.

Supplemental references

Arora C., K. Kee, S. Maleki, and S. Keeney, 2004 Antiviral protein Ski8 is a direct partner of Spo11 in meiotic DNA break formation, independent of its cytoplasmic role in RNA metabolism. Mol. Cell 13: 549–559.

Bolt M. W., and P. A. Mahoney, 1997 High-efficiency blotting of proteins of diverse sizes following sodium dodecyl sulfate-polyacrylamide gel electrophoresis. Anal. Biochem. 247: 185–192.

Chu D. B., T. Gromova, T. A. C. Newman, and S. M. Burgess, 2017 The Nucleoporin Nup2 Contains a Meiotic-Autonomous Region that Promotes the Dynamic Chromosome Events of Meiosis. Genetics 206: 1319–1337.

Haar T. von der, 2007 Optimized protein extraction for quantitative proteomics of yeasts. PLoS One 2: e1078.

Holzer G., P. De Magistris, C. Gramminger, R. Sachdev, A. Magalska, *et al.*, 2021 The nucleoporin Nup50 activates the Ran guanine nucleotide exchange factor RCC1 to promote NPC assembly at the end of mitosis. EMBO J. 40: e108788.

Jumper J., R. Evans, A. Pritzel, T. Green, M. Figurnov, *et al.*, 2021 Highly accurate protein structure prediction with AlphaFold. Nature 596: 583–589.

Mariani V., M. Biasini, A. Barbato, and T. Schwede, 2013 lDDT: a local superposition-free score for comparing protein structures and models using distance difference tests. Bioinformatics 29: 2722–2728.

Pettersen E. F., T. D. Goddard, C. C. Huang, E. C. Meng, G. S. Couch, *et al.*, 2021 UCSF ChimeraX: Structure visualization for researchers, educators, and developers. Protein Sci. 30: 70–82.

Saiz-Baggetto S., E. Méndez, I. Quilis, J. C. Igual, and M. C. Bañó, 2017 Chimeric proteins tagged with specific 3xHA cassettes may present instability and functional problems. PLoS One 12: e0183067.
